# Auto-antibody evaluation in idiopathic interstitial pneumonia and worse survival of patients with Ro52/TRIM21auto-antibody

**DOI:** 10.3164/jcbn.20-5

**Published:** 2020-05-15

**Authors:** Hiroko Morita, Yasuo Shimizu, Yusuke Nakamura, Hiroaki Okutomi, Taiji Watanabe, Tatsuya Yokoyama, Sayo Soda, Naoya Ikeda, Taichi Shiobara, Masaaki Miyoshi, Kazuyuki Chibana, Akihiro Takemasa, Kazuhiro Kurasawa

**Affiliations:** 1 Department of Pulmonary Medicine and Clinical Immunology, Dokkyo Medical University School of Medicine, 880 Kitakobayashi, Mibu, Tochigi 321-0293, Japan; 2 Department of Rheumatology, Dokkyo Medical University School of Medicine, 880 Kitakobayashi, Mibu, Tochigi 321-0293, Japan

**Keywords:** Ro52, TRIM21, interstitial pneumonia, myositis panel, auto-antibody

## Abstract

Some patients with interstitial pneumonia (IP) have auto-antibodies, but do not fit the criteria for specific connective tissue diseases. Examination of auto-antibodies is recommended for diagnosis idiopathic pulmonary fibrosis. A prospective cohort study was performed in 285 patients with IP. Eleven auto-antibodies were assessed and patients were followed for 2 years. All 285 patients underwent the myositis panel test (MPT) for 11 auto-antibodies. Among them, 23.5% (67/285) of the patients had a positive MPT and 14.7% (42/285) had connective tissue diseases. Among the 49 MPT positive patients without connective tissue diseases, 29 patients (59.2%) were positive for Ro52, including 17 patients with Ro52 mono-positivity. Among interstitial pneumonia patients without connective tissue diseases, the Ro52 mono-positive patients showed worse at 2-years survival than those who were Ro52 negative (*p* = 0.022, HR = 5.88, 95% CI 1.29–26.75). Most of the Ro52 positive patients also showed a low titer of anti-nucleolar antibody. About 20% of IP patients had auto-antibodies detectable by the MPT, and Ro52 positive patients accounted for more than half of the MPT positive patients without connective tissue diseases. Detection of Ro52 auto-antibodies may be useful for assessing the risk of progression in idiopathic interstitial pneumonia patients without connective tissue diseases and a low anti-nucleolar antibody titer.

## Introduction

Many diseases are associated with interstitial pneumonia (IP) and the causes of this condition are varied.^(1,2)^ Idiopathic interstitial pneumonia (IIP) is defined as a subset of the group of subacute and chronic lung disorders collectively referred to as IP diseases or diffuse parenchymal lung diseases of unknown aetiology.^(1,3)^ Diagnosis of IIP is confirmed by performing appropriate clinical, radiological, and surgical examinations to exclude secondary IP. Some IP patients have autoimmune features that do not fit the criteria for diagnosis of any specific connective tissue disease (CTD), so IIP is diagnosed.^(4)^ It has been proposed that IP with autoimmune features should be classified as undifferentiated connective tissue diseases (UCTD),^(5,6)^ lung-dominant CTD,^(7)^ autoimmune-featured interstitial lung disease (AIF-ILD),^(8)^ or IP with autoimmune features (IPAF).^(9)^

Serological tests for auto-antibodies are often helpful during initial evaluation of IP,^(10–12)^ and auto-antibody positivity is one of the proposed criteria for IP with autoimmune features. Although various novel auto-antibodies have been discovered, it is not always possible to measure these in the clinical setting,^(13,14)^ but a commercial assay recently became available for some new auto-antibodies.^(15,16)^ Measuring these auto-antibodies in patients who have IP with autoimmune features could clarify differences between IIPs and CTD-IP, and could contribute to improving the proposed criteria for IP with autoimmune features. In addition, serological tests such as the myositis panel have been recommended for making a diagnosis of idiopathic pulmonary fibrosis in an official American Thoracic Society, European Respiratory Society, Japanese Respiratory Society, and Latin American Thoracic Society clinical practice guideline 2018.^(17)^

In the present study, immunoassay for auto-antibodies was performed in IP patients by using a myositis panel test (MPT) kit, which provides quantitative serum levels of IgG auto-antibodies targeting the following 11 antigens: Mi-2β (helicase protein, part of the NuRD complex), Ku (thyroid autoantigen), PM-scl100, PM-scl75, Jo-1 (histidyl-tRNA synthetase), SRP (signal recognition particle, ribonucleoprotein complex), PL-7 (threonyl-tRNA synthetase), PL-12 (alanyl-tRNA synthetase), EJ (glycyl-tRNA synthetase), OJ (isoleucil-tRNA synthetase), and Ro52 (tripartite motif 21, TRIM21).^(15)^ Anti-aminoacyl-tRNA synthase (ARS), including anti-Jo-1, anti-PL-7, anti-PL-12, anti-EJ, anti-KS, and anti-OJ antibodies, can be measured with other commercial assays, but not anti-Mi-2β, -Ku, -PM-Scl75, -SRP, and -Ro52 antibodies. Among these antigen, Ro52 ubiquitilates p62/sequestosome1 (SQSTM1) and supresses protein sequestration to regulate redox homeostasis, and previously we reported that p62/SQSTM1 was co-localized to iron binding silica with oxidative stress lung in mice.^(18,19)^

The present study was performed to clarify the positive rates of 11 different auto-antibodies in IP patients. In addition, after excluding IP patients who met the criteria for diagnosis of CTDs, survival of the remaining anti-Ro52 positive IP patients was investigated.

## Methods

### Subjects

A total of 285 patients with a diagnosis of IP based on chest computed tomography (CT) were enrolled. Patients were excluded if they were under 20 years old. CTDs were diagnosed by department of Rheumatology according to the reported criteria,^(20–27)^ and patients in whom CTDs were diagnosed were excluded from further analysis. IP was evaluated according to the Official American Thoracic Society/European Respiratory Society Statement: Update of the International Multidisciplinary Classification of the Idiopathic Interstitial Pneumonias,^(3)^ with chest CT scans being reviewed by three experienced chest physicians who were blinded to symptoms and laboratory test results. Progression of IP or respiratory failure to death was defined as lung-related death. This study was carried out in accordance with the Declaration of Helsinki and informed consent was obtained from the patients. The study was approved by the Human Research Committee of Dokkyo Medical University (no. 2114 and R-9-2), and was registered with the University Hospital Medical Information Network (UMIN 32926).

### Blood sampling and analysis

Serum obtained from a peripheral blood sample was stored at –80°C until analysis. Measurement of the anti-nuclear antibody (ANA) titer was carried out by the fluorescent antibody technique (FA), and a titer ≤40 was defined as negative for ANA according to the manufacturer’s directions (SRL, Inc., Hachioji, Tokyo, Japan). Antibodies for Mi-2β, Ku, PM-scl100, PM-scl75, Jo-1, SRP, PL-7, PL-12, EJ, OJ, and Ro52 were detected by using a line immunoassay [EUROLINE Myositis Antigen Profile 3 (IgG) test (MPT); EUROIMMUN, Lubeck, Germany], and a positive result was defined as 2+ according to the manufacturer’s information.

### Statistical analysis

Differences between two groups were calculated by using the chi-square test. For comparison of continuous variables, the Mann-Whitney *U* test was used. Kaplan-Meier analysis was employed to compare survival between groups and the odds ratio (OR) was calculated with the 95% confidence interval (CI). *P* value <0.05 was considered significant, and *p*<0.1 indicated a trend.

## Results

**Fig. 1:**
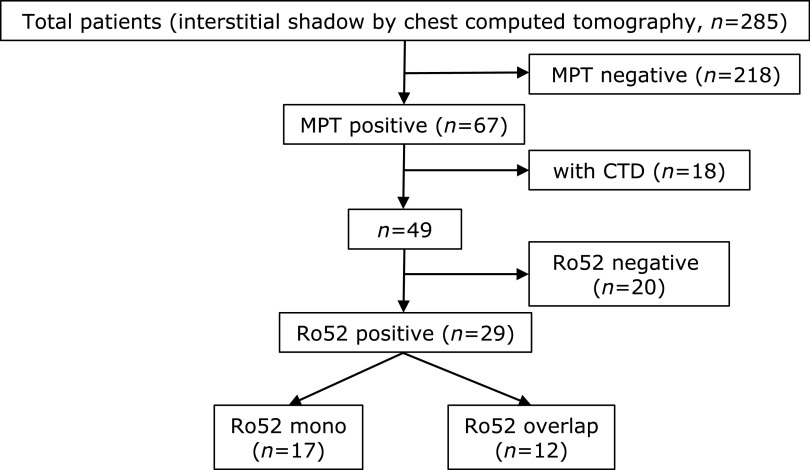
Study protocol and number of patients enrolled. MPT, myositis panel test; CTDs, connective tissue diseases. Bold arrows indicate main flow of this study.

An outline of the study and the number of patients enrolled are shown in Fig. 1. A total of 285 patients underwent the MPT, with 67 patients (23.5%) being positive and 218 patients (76.5%) being negative. Characteristics of all enrolled patients are summarized in Supplemental Table 1*. The gender ratio and smoking status were significantly different between MPT positive and MPT negative patients (*p*<0.05). In both MPT positive and negative patients, chest CT showed fibrotic-nonspecific IP (f-NSIP) and usual IP (UIP) more often than other patterns, but OP was also frequently observed in MPT negative patients.

After extensive workup to detect CTDs, 18 MPT positive patients and 24 MPT negative patients were diagnosed as having CTDs. After excluding these CTD patients, 49 MPT positive and 194 MPT negative patients underwent further analysis (Fig. 1, Table 1). The characteristics of the MPT positive patients (49/243, 20.2%) and MPT negative patients (194/243, 79.8%) were similar to those before excluding the CTD patients (Supplemental Table 1* and Table 1).

Based on the MPT, patients were divided into three groups: Ro52 mono-positive (Ro52 mono), positive for Ro52 and other antibodies (Ro52 overlap), and Ro52 negative but positive for other antibodies (Ro52 negative) (Fig. 1). In all 3 groups, most patients had no symptoms related to CTDs, but several patients presented with CTD-like symptoms (Table 2). Edema of the extremities was most often observed in the Ro52 mono group.

**Fig. 2:**
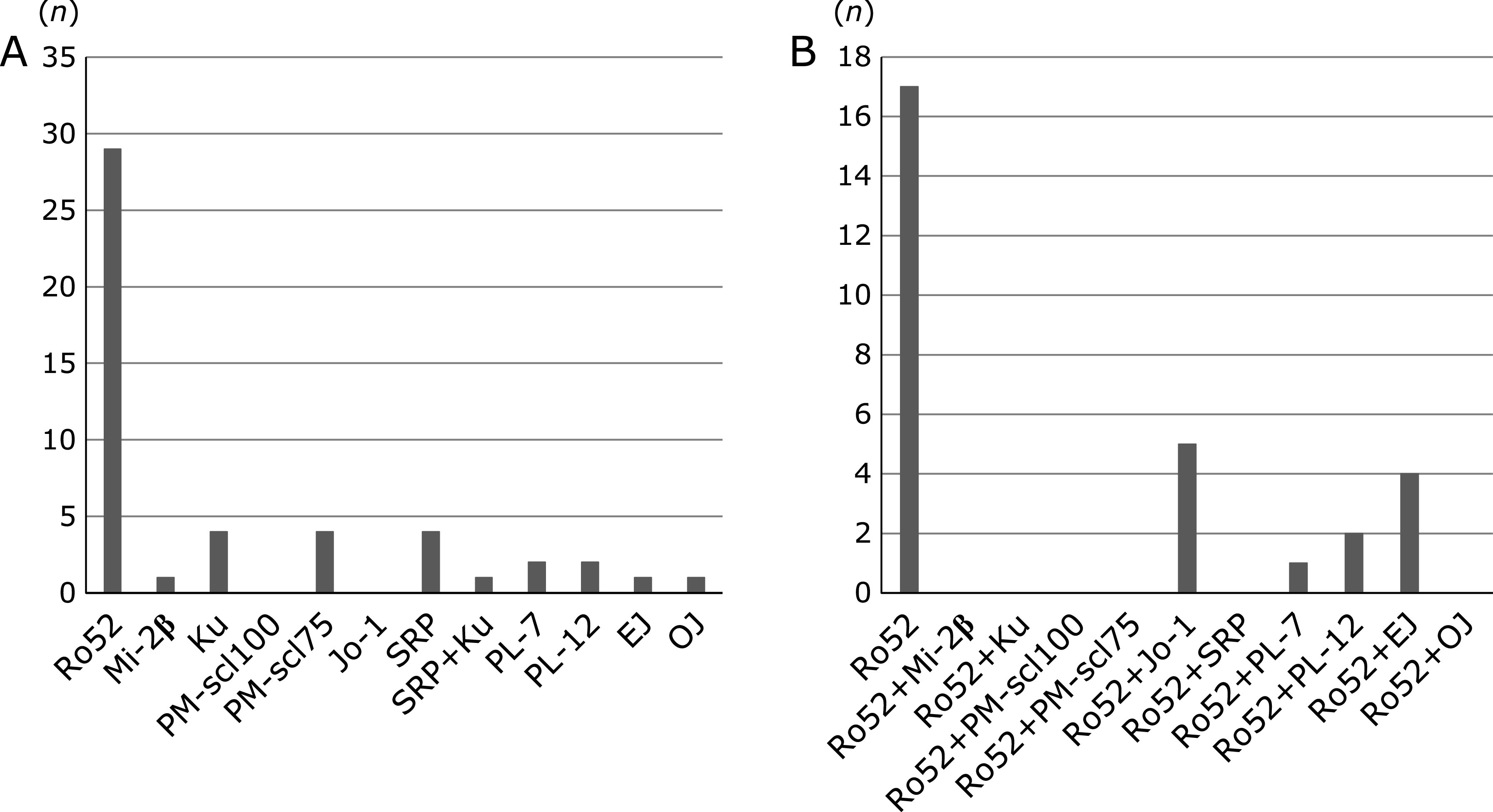
(A) Number of patients positive for auto-antibodies in the myositis panel test (MPT). MPT positive IP patients without CTDs were investigated (*n* = 49). (B) Number of patients with Ro52 mono-positivity and Ro52 overlap in the MPT. Ro52 positive IP patients without CTDs were investigated (*n* = 29).

Of the 49 MPT positive patients, 29 patients (29/49, 59.2%) were Ro52 positive (Ro52 mono and Ro52 overlap) and 20 patients were Ro52 negative (Fig. 2A). Among Ro52 negative patients, antibodies for Ku, scl-PM75, and SRP were relatively frequent compared to other antibodies. Of the 29 Ro52 positive patients, 17 patients were in the Ro52 mono group (17/29, 58.6%) and 12 were in the Ro52 overlap group (12/29, 41.4%) (Fig. 2B). In the Ro52 overlap group, positivity for Ro52 + Jo-1 and Ro52 + EJ was relatively frequent.

**Fig. 3:**
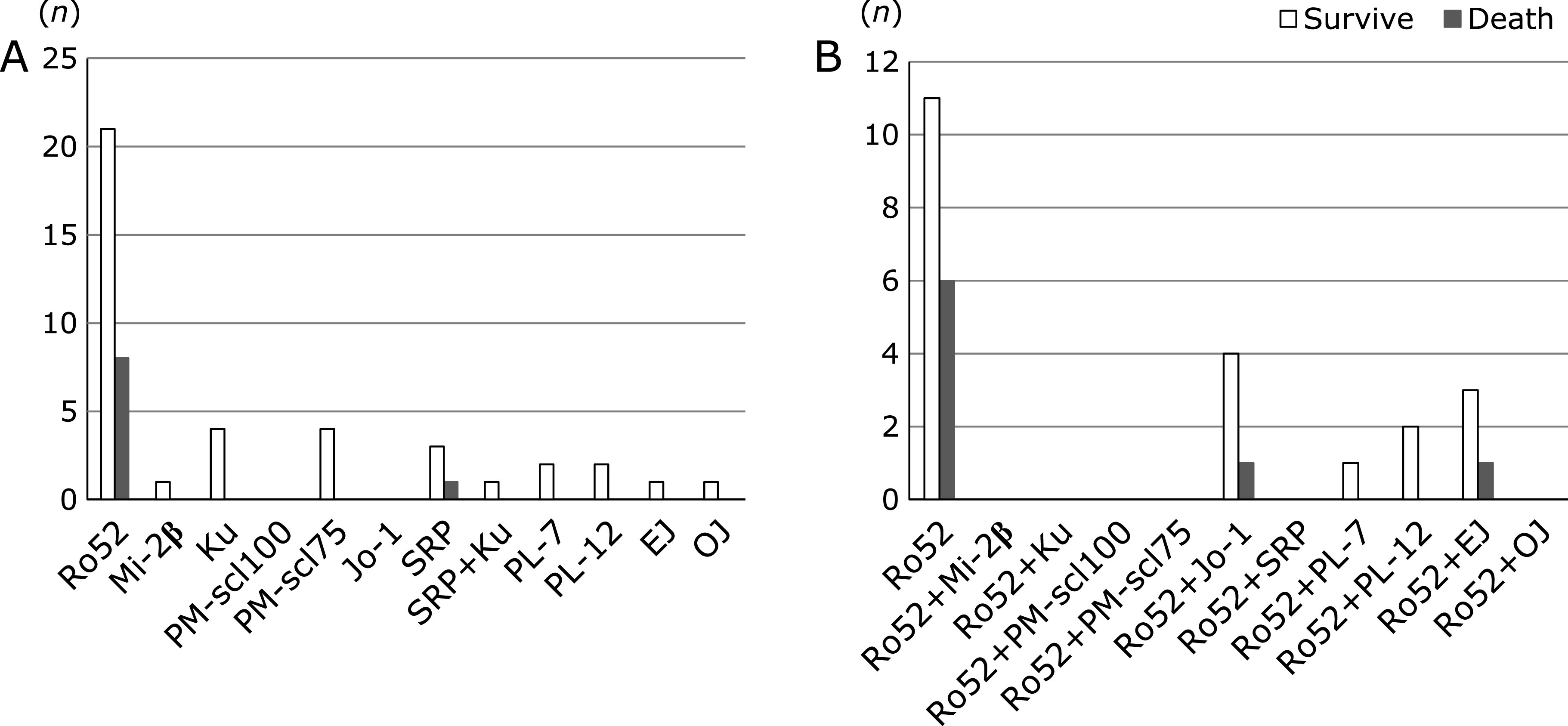
(A) Positivity for auto-antibodies and lung-related death in the myositis panel test (MPT). MPT positive IP patients without CTDs were investigated (*n* = 49). The observation period was 24 months. (B) Lung-related death among Ro52 positive patients. Ro52 positive IP patients without CTDs were investigated (*n* = 29). The observation period was 24 months.

**Fig. 4:**
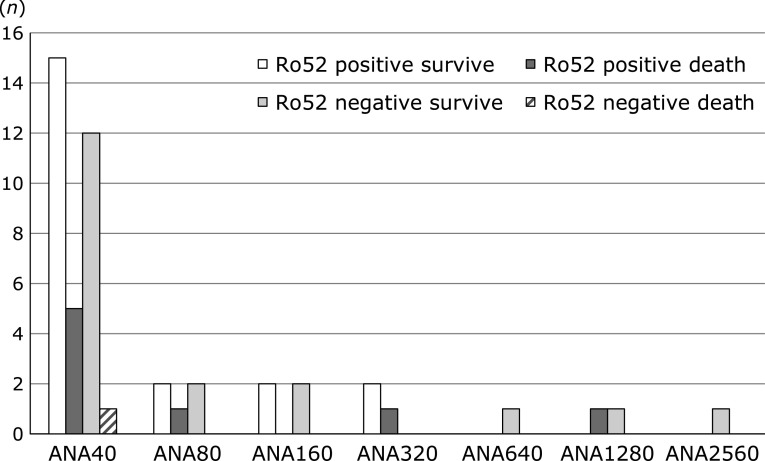
Lung-related death among patients positive for auto-antibodies in the myositis panel test (MPT) stratified by the ANA titer. MPT-positive IP patients without CTDs were investigated (*n* = 49). The observation period was 24 months. White: surviving Ro52 positive patients, black: Ro52 positive patients who died, mesh: Ro52 negative patients who were positive for other antibodies and survived, diagonal lines: Ro52 negative patients who were positive for other antibodies and died.

Lung-related death occurred in 8/29 (27.6%) Ro52 positive patients and 1/20 (5%) Ro52 negative patients, with lung-related death showing a significantly higher frequency among Ro52 positive patients than Ro52 negative patients (*p* = 0.045) (Fig. 3A). Among Ro52 positive patients (*n* = 29), lung-related death occurred in 6/17 (35.3%) patients in the Ro52 mono group (*n* = 17), as well as in 1/5 (20.0%) Ro52 + Jo-1 patients and 1/4 (25%) Ro52 + EJ patients, but in none of the other overlap patients (*n* = 3) (Fig. 3B). The lung-related death rate was not statistically different between the Ro52 mono and Ro52 overlap groups. Analysis of lung-related death stratified by the ANA titer was done in Ro52 positive and negative patients (Fig. 4). When ANA was ≤40, lung-related death occurred in 5/20 (25%) Ro52 positive patients vs 1/13 (7.7%) Ro52 negative patients. When ANA was ≥80, lung-related death occurred in 3/9 (33.3%) Ro52 positive patients vs 0/7 (0%) Ro52 negative patients. There was no significant difference of lung-related death between Ro52 positive and negative patients with ANA ≤40, or between Ro52 positive patients with ANA ≤40 or ANA ≥80. Analysis of lung-related death stratified by the ANA titer was also done in Ro52 positive patients, comprising the Ro52 mono and Ro52 overlap groups, but there was no significant difference between these two groups (data not shown).

The background of MPT positive patients with lung-related death (*n* = 8) is shown in Table 3. Six of the eight patients were in the Ro52 mono group. The number of male and female patients was similar. On chest CT, f-NSIP was the most frequent pattern and UIP was next, but some patients showed cellular-NSIP (c-NSIP) and acute IP (AIP). Most patients had a low ANA titer, although two patients showed a high titer. Six patients presented with acute exacerbation and two patients had chronic progressive IP.

**Fig. 5:**
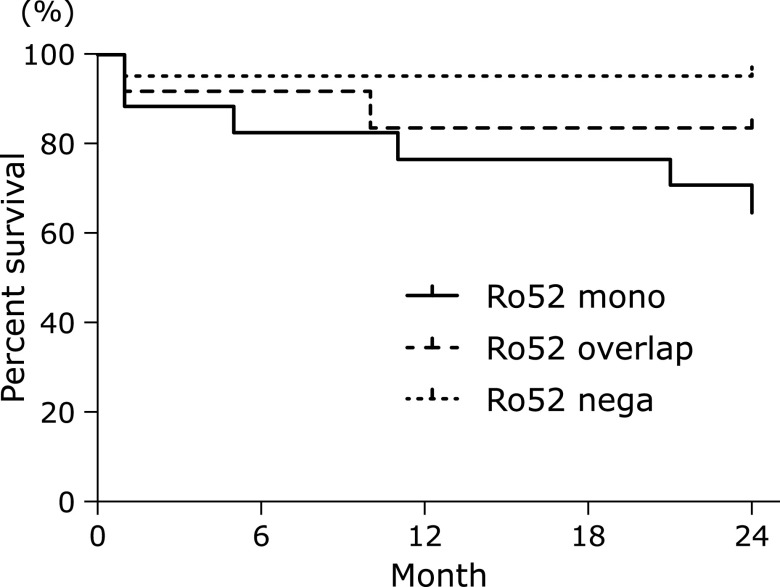
Kaplan-Meier curves comparing survival of the Ro52 mono-positive group (Ro52mono, *n* = 17), Ro52 overlap group (Ro52overlap, *n* = 12), and Ro52 negative group (Ro52nega, *n* = 20) up to 24 months. IP patients without CTDs who were positive in the myositis panel test (MPT) were investigated (*n* = 49).

When 2-year survival was compared between Ro52 positive and negative patients, the Ro52 positive patients had an increased risk of death relative to Ro52 negative patients (*p* = 0.0518, HR = 3.78, 95% CI 0.99–14.45) (Supplemental Fig. 1*). Next, 2-year survival was compared among the Ro52 mono, Ro52 overlap, and Ro52 negative groups. The Ro52 mono group showed significantly worse survival than the Ro52 negative group (*p* = 0.022, HR = 5.88, 95% CI 1.29–26.75), but there was no statistical difference compared with the Ro52 overlap group (Fig. 5). The ratio for the patients receiving immunosuppressive agents were not different among the groups.

## Discussion

This study revealed that anti-Ro52 monospecific positive patients without CTDs had worse 2-year survival than Ro52 negative patients, even though most of them showed a low ANA titer.

CTDs are a major cause of IP in the USA, accounting for about 20% of patients, while a multi-ethnic study performed in Paris showed that CTDs occurred in about 16% of IP patients.^(28,29)^ In present study, CTDs were found in 14.7% (42/285) of IP patients. Since this study was performed to investigate patients with serological autoimmune features that did not fulfill the criteria for established CTDs, the 42 CTD patients were excluded from analysis (their characteristics are shown in Supplemental Table 2*).

In the absence of defined CTDs, 10 to 20% of IIPs patients have been reported to show serological abnormalities.^(5)^ After excluding IP patients with CTDs from the study population, 20.2% (49/243) of the remaining IP patients were MPT positive. A previous investigation of the prevalence of auto-antibodies revealed MPT positivity in a high 37.5% (12/32) of IIP patients.^(30)^ Possible reasons for the difference from the present study include a high ANA positive rate of 66.7% (8/12) among MPT positive patients in the other study and its small patient population.^(30)^ Another investigation of these antibodies showed that anti-Ro52 was not only frequently detected in idiopathic inflammatory myositis, but was also prevalent in other CTDs.^(31)^ In patients with systemic sclerosis (SSc), Ro52 positivity was associated with concomitant IP and with worse survival.^(32)^ However, the prognosis of patients with IIPs and Ro52 auto-antibody positivity has not been investigated.

Among the 11 auto-antibodies investigated in this study, Ro52 was detected most frequently. Among the Ro52 positive patients, patients with Ro52 mono-positivity were more frequent (35.3%) than those with other auto-antibodies. According to the literature, the prevalence of Ro52 mono-positive patients with IP accompanied by CTDs is 50% among patients with mixed connective tissue disease (MCTD),^(33)^ 80% among those with idiopathic inflammatory myopathies,^(31)^ 57% or 36.2% among those with SSc,^(32,34)^ and 5.6% among those with UCTD.^(35)^ Healthy subjects were not positive for Ro52 in another study.^(13)^ Based on these reports, the frequency of Ro52 mono-positivity seems to be lower among non-CTD patients with IP than among patients with established CTDs and IP, but higher than among patients with UCTD and IP.

In Ro52 positive patients with SSc, symptoms other than IP showing a higher frequency than in Ro52 negative patients were reported to be fecal incontinence, hyperalimentation, gastroesophageal symptoms, and pulmonary hypertension.^(30)^ In the present study, edema of the extremities was most frequent in the Ro52 mono and Ro52 negative groups. This result might have been obtained because edema was easy to detect during examination and because Ro52 positivity reflects systemic edema.^(36,37)^ It was reported that Ro52 positivity is not specific to myositis and is more prevalent in other CTDs.^(16)^ This could be another reason for the lack of specific symptoms other than IP among Ro52 positive patients.

There have been no reports about the chest CT features of IIP on MPT positive or Ro52 positive patients. Although it was reported that among Norwegian patients with MCTD, Ro52 mono-positive patients more frequently showed severe pulmonary fibrosis than Ro52 negative patients, the detailed CT findings were not described.^(33)^ Among seven IPAF patients who developed acute respiratory distress syndrome (ARDS), it was reported that six patients were Ro52 positive. Of these six patients, CT scans obtained before the onset of ARDS revealed OP in four, acute IP/diffuse alveolar damage (AIP/DAD) in one, and lymphatic interstitial pneumonia (LIP) in one.^(38)^ In present study, f-NSIP was the most frequent pattern and UIP was next, but some patients showed cellular-NSIP (c-NSIP) and acute IP (AIP). Patients with OP and c-NSIP are considered to show a better response to corticosteroids or immunosuppressive agents than f-NSIP and UIP.^(39)^ However, if patients are Ro52 positive, it seems important to be aware of the possibility of a poor response to treatment and the elevated risk of lung-related death. A previous study of SSc revealed that Ro52 positive patients with IP showed worse survival after 40 months, especially Ro52 mono-positive patients.^(32)^ In the present study, there was no statistical difference of survival between the Ro52 mono and Ro52 overlap groups up to 2 years, but the Ro52 mono group had significantly worse survival than the Ro52 negative group. The reason for this difference of survival between Ro52 mono-positive patients and Ro52 overlap patients is unknown. The number of patients studied might have affected the results obtained or co-existence of other auto-antibodies could influence the disease phenotype.^(32,40,41)^

There have been no investigations of the association between lung-related death and the ANA titer in non-CTD Ro52 positive or negative patients with IP. The number of lung-related deaths was highest among Ro52 positive patients with ANA ≤40, but the lung-related death rate showed no statistical difference in relation to the ANA titer. Ro52 is abundant in the cytoplasm, and this could lead to a negative result for ANA by fluorescent antibody testing in Ro52 positive patients. Based on our findings, it is important to carefully manage non-CTD Ro52 positive patients with IP and any ANA titer, and those who are ANA negative but Ro52 positive also have a risk of developing severe IP. In the present study, a patient who was anti-SRP positive but Ro52 negative also developed IP and died. It was reported that anti-SRP positive patients were less likely to be complicated by IP than patients with other myositis-related auto-antibodies,^(31,42)^ but some of these patients developed severe IP in other studies.^(43,44)^

Inhibition of Ro52 signaling has been proposed to be involved in induction of IL-17 production and inflammation.^(45)^ Ro52 possesses RING-dependent E3-ubiquitine ligase activity and negatively regulates the production of pro-inflammatory cytokines, and also plays an essential role in p62/SQSTM1-regulated redox homeostasis.^(18,45)^ In addition, binding of IgG Fc to the PRY-SPRY domain allows Ro52 to act as a cytoplasmic Fc antibody receptor.^(46–48)^ In mice, Ro52 knockout induces tissue inflammation and systemic autoimmunity via upregulation of the IL-23/IL-17 pathway.^(37)^ In patients with inflammatory bowel disease, expression of Ro52 is reduced in CD4+ T cells infiltrating the inflamed mucosa.^(49)^ Enhancement of Th1/Th17 inflammation by inhibition of Ro52 was also proposed to have a role in Beçhet disease.^(50)^ Nothing has been reported about lung inflammation in Ro52 knockout mice or humans. 1, 25-dihydroxycholecalciferol calcitriol, [1,25(OH)_2_D_3_] is activate form of vitamin D, and calcitriol inhibited IL-23/IL-17 in the airway of cystic fibrosis patients.^(51)^ Furthermore, calcitriol prevented experimental lung fibrosis model in mice via inhibiting inflammation and accumulation of activated fibroblasts.^(52)^ Thus, IL-23/IL-17 pathway possibly is involved in IP development, however our preliminary examination of the serum IL-17 concentration did not show apparent elevation of IL-17A in Ro52 positive patients. To further investigate the contribution of Th17-based inflammation to IP, it would be necessary to examine lung tissue specimens.

There were several limitations of the present study. Treatment for IP depended on the attending doctors, although corticosteroids and/or immunosuppressive agents were used to treat all of the patients with lung-related death.

In conclusion, Ro52 positive IP patients without CTDs had worse 2-year survival than Ro52 negative patients, even though most of them showed a low ANA titer. Therefore, these patients require careful observation to detect progression of IP and allow initiation of medication with appropriate timing.

## Author Contributions

HM undertook data collection, contributed to discussion the results and wrote the manuscript. YS designed the study, undertook data collection, contributed to discussion the results and wrote the manuscript. KK designed the study and contributed to discussion the results. YN, HO, TW, TY, SS, TS, KC undertook data collection and treat patients. NI, MM, and AT determine the CT patterns.

## Figures and Tables

**Table 1:** Characteristics of patients without connective tissue diseases

Parameters	MPT positive	MPT negative
Total (*n*)	49 (20.2%)	194 (79.8%)

Gender (*n*)		
Male	28	125
Female	21*	69

Age [mean (SD)]	70.3 (10.7)	70.2 (11.0)

Smoking status (*n*)		
Former, Current	25	118
Never	24*	76
Pack-year [mean (SD)]	29.3 (42.1)	25.7 (37.4)

HRCT pattern (*n*)		
UIP	13	63
f-NSIP	22	75
c-NSIP	8	12
AIP	3	8
COP	0	27
PPFE	0	4
RB-ILD	0	2
HP	0	3

Data are presented as mean ± SD and number of patients as *n*. MPT, myositis panel test. HRCT pattern are indicated usual interstitial pneumonia (UIP), fibrotic non-specific interstitial pneumonia (f-NSIP), cellular non-specific interstitial pneumonia (c-NSIP), acute interstitial pneumonia (AIP), cryptogenic organizing pneumonia (OP), pleuroparenchimal fibroelastosis (PPFE), respiratory bronchiolitis-associated interstitial lung disease (RB-ILD), and hypersensitivity pneumonia (HP). *p<0.05.

**Table 2:** Clinical symptoms of patients in myositis panel test positive patients

Clinical symptoms related to connective tissue diseases	Ro52 mono group (*n* = 17)	Ro52 overlap group (*n* = 12)	Ro52 negative group (*n* = 20)
Symptoms positive	3	2	5
Edema of extremities	3	0	1
Dry mouth	0	0	0
Muscle weakness	1	0	1
Mechanic hand	1	0	0
Pleural effusion	0	0	0
Raynaud	0	0	0
Pericardial effusion	0	0	0
Fever	0	0	1
Neck pain	0	1	0
Erythema	0	0	2
Dry eye	0	1	0
Multiple joint edema	0	0	0
Shoulder poikiloderma	0	0	0
Joint stiffness	0	0	3
Myalgia	0	1	0
Symptoms negative	14	10	15

Number of patients are presented as *n*. Ro52 mono, Ro52 mono positive in myositis panel test (MPT) patients; Ro52 overlap, Ro52 positive and other antibodies overlap in MPT; Ro52 negative, Ro52 negative but other antibody positive in MPT.

**Table 3:** Characteristics of patients of lung related death in myositis test positive

Patient	M/F	CT	ANA titer	Clinical symptoms related to connective tissue disease
Ro52 mono				
Pt1	M	UIP	320 (speckled)	None
Pt2	M	f-NSIP	≤40	Muscle weakness, erythema of PIP with edema
Pt3	M	f-NSIP	1,280 (speckled)	None
Pt4	F	c-NSIP	≤40	None
Pt5	F	AIP	80	None
Pt6	F	f-NSIP	≤40	None

Ro52 overlap				
pt7 (Ro52 + EJ)	M	UIP	≤40	None
pt8 (Ro52 + Jo1)	M	UIP	≤40	None

Pt, patient; M/F, male/female; ANA, antinuclear antigen; PIP, proximal interphalangeal joint.
