# Supplementary material for: Auto-antibody evaluation in idiopathic interstitial pneumonia and worse survival of patients with Ro52/TRIM21auto-antibody
Source: J Clin Biochem Nutr. 2020 May 15;67(2):199–205. doi: 10.3164/jcbn.20-5 (PMC7533866; doi:10.3164/jcbn.20-5)
Supplement: Supplemental Figure 1 [file jcbn20-5sf01.pdf]

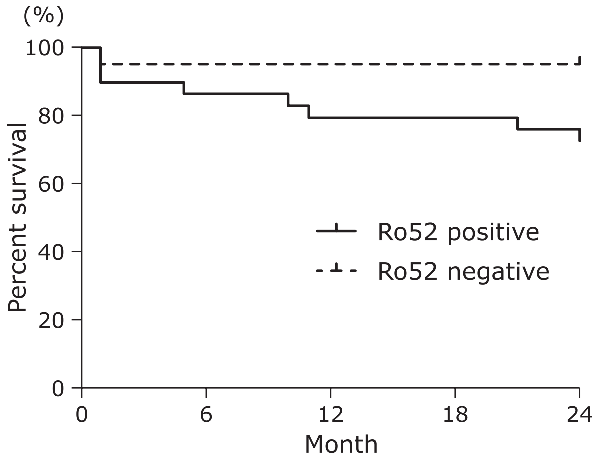

Supplemental Fig. 1. Kaplan-Meier curve to compare survival in the Ro52 positive patients (n = 29) and Ro52 negative but other antibodies positive patients (n = 20) for 24 months period. IP patients showing myositis panel test (MPT) positive without CTDs were investigated (total n = 49).
