# Supplementary material for: Auto-antibody evaluation in idiopathic interstitial pneumonia and worse survival of patients with Ro52/TRIM21auto-antibody
Source: J Clin Biochem Nutr. 2020 May 15;67(2):199–205. doi: 10.3164/jcbn.20-5 (PMC7533866; doi:10.3164/jcbn.20-5)
Supplement: Supplemental Table 1 [file jcbn20-5st01.pdf]

Supplemental Table 1. Characteristics of enrolled patients in this study

Parameters	MPT positive	MPT negative
Total (*n*)	67 (23.5%)	218 (76.4%)
Gender (*n*)		
Male	31	141
Female	36*	77
Age [mean (SD)]	69.7 (10.9)	69.7 (10.8)
Smoking status (*n*)		
Former, Current	27	164
Never	40*	54
Pack-year [mean (SD)]	23.5 (39.6)	25.8 (36.6)
HRCT pattern (*n*)		
UIP	18	75
f-NSIP	31	81
c-NSIP	9	15
AIP	6	8
COP	3	29
PPFE	0	4
RB-ILD	0	3
HP	0	3

Data are presented as mean ± SD and number of patients as *n*. MPT, myositis panel test. HRCT classification are indicated usual interstitial pneumonia (UIP), fibrotic non-specific interstitial pneumonia (f-NSIP), cellular non-specific interstitial pneumonia (c-NSIP), acute interstitial pneumonia (AIP), cryptogenic organizing pneumonia (OP), pleuroparenchimal fibroelastosis (PPFE), respiratory bronchiolitis-associated interstitial lung disease (RB-ILD), and hypersensitivity pneumonia (HP). *$p<0.05$.
