# Supplementary material for: Auto-antibody evaluation in idiopathic interstitial pneumonia and worse survival of patients with Ro52/TRIM21auto-antibody
Source: J Clin Biochem Nutr. 2020 May 15;67(2):199–205. doi: 10.3164/jcbn.20-5 (PMC7533866; doi:10.3164/jcbn.20-5)
Supplement: Supplemental Table 2 [file jcbn20-5st02.pdf]

Supplemental Table 2. Number of patients of connective tissue diseases with myositis panel test positive and the each antibody

Diagnosis for CTDs	Number of CTDs patients	Total Ro52 positive	Myositis panel test positive number of patients (antibody)			CT number of patients (pattern)		
			Ro52 mono	Ro52 overlap	Ro52 negative	Ro52 mono	Ro52 overlap	Ro52 negative
PM	2	1	0	1 (Jo-1)	1 (Mi-2β)	0	1 (f-NSIP)	1 (AIP)
DM	3	1	0	1 (SRP)	2 (Ku, PM-scl75)	0	1 (UIP)	1 (fNSIP), 1 (UIP)
PM/DM	1	1	1	0	0	1 (AIP)	0	0
SjS	7	4	2	2 (PL-12, EJ)	3 (PM-scl75, EJ, Ku)	2 (f-NSIP)	2 (f-NSIP)	2 (f-NSIP), 1 (UIP)
SjS + PM/DM	1	0	0	0	1 (Jo-1)	0	0	1 (f-NSIP)
SjS + SSc	1	1	0	1 (PM-scl75)	0	0	1 (UIP)	0
RA	2	2	2	0	0	1 (AIP), 1 (f-NSIP)	0	0
ANCA vasculitis	1	0	0	0	1 (Mi-2β)	0	0	1 (c-NSIP)
Total (*n*)	18	10	5	5	8			

CTDs, connective tissue diseases; MPT, myositis panel test. Number of patient (each MPT antibody or chest CT pattern) are indicated. Ro52mono, Ro52 mono positive in myositis panel test (MPT) patients; Ro52overlap, Ro52 positive and other antibodies overlap in MPT; Ro52 negative, Ro52 negative but other antibody positive in MPT. HRCT patterns are indicated usual interstitial pneumonia (UIP), fibrotic non-specific interstitial pneumonia (f-NSIP), cellular non-specific interstitial pneumonia (c-NSIP), acute interstitial pneumonia (AIP), cryptogenic organizing pneumonia (OP), pleuroparenchimal fibroelastosis (PPFE), respiratory bronchiolitis-associated interstitial lung disease (RB-ILD), and hypersensitivity pneumonia (HP).
